# Four-branched hybrid stent placement using multi-hole-covered self-expandable metallic stents for Bismuth type IV malignant hilar biliary obstruction

**DOI:** 10.1055/a-2762-8106

**Published:** 2026-01-15

**Authors:** Shinya Kawaguchi, Shinya Endo, Tatsunori Satoh

**Affiliations:** 1 26389 Department of Gastroenterology, Shizuoka General Hospital, Shizuoka, Japan

**Fig. 1:**
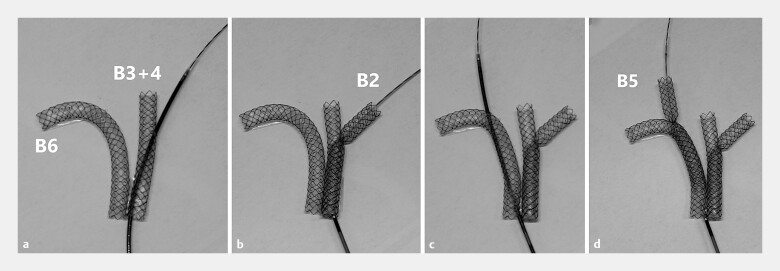
Bench-top photograph demonstration of hybrid stent placement combining side-by-side (SBS) and stent-in-stent (SIS) techniques using multi-hole-covered self-expandable metallic stents (MHSEMSs). **a** The first and second MHSEMSs were deployed into B3 + 4 and B6 in an SBS configuration, followed by the preparation of the third-branch insertion through the side hole. **b** The third MHSEMS was inserted into B2 using the SIS technique through the side hole of the left stent. **c** Preparation of the fourth-branch insertion through the side hole. **d** The fourth MHSEMS was inserted into B5 using the SIS technique through the side hole of the right stent, completing the four-branched hybrid stenting.

Hybrid stent placement during endoscopic retrograde cholangiopancreatography (ERCP), combining side-by-side (SBS) and stent-in-stent (SIS) techniques allows multi-segmental drainage of complex malignant hilar biliary obstruction (MHBO 1 2 ). Conventionally, uncovered self-expandable metallic stents (SEMSs) are used. A multi-hole-covered SEMS (MHSEMS; HANAROSTENT Biliary Multi-hole Benefit; M.I. Tech Co., Ltd, Pyeongtaek, South Korea) has recently been developed, designed with side holes to facilitate branch access, prevent side-branch occlusion, tumour ingrowth, and stent migration, while maintaining removability 3 4 5 . Here, we describe the first case of hybrid SBS and SIS placement using four MHSEMSs, achieving four-branched drainage in a bismuth type IV MHBO ( Fig. 1 ).

**Fig. 2:**
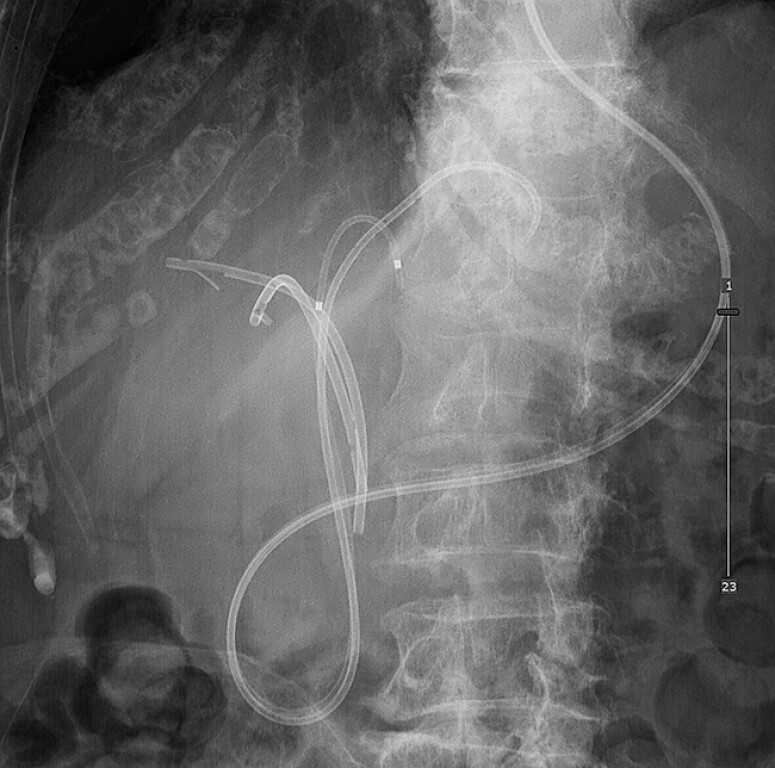
A fluoroscopic image before the current procedure showing two inside plastic stents placed at B5 (right anterior) and B6 (right posterior) ducts, and two 5 Fr endoscopic nasobiliary drainage tubes placed in B3 + 4 and B2.

**Fig. 3:**
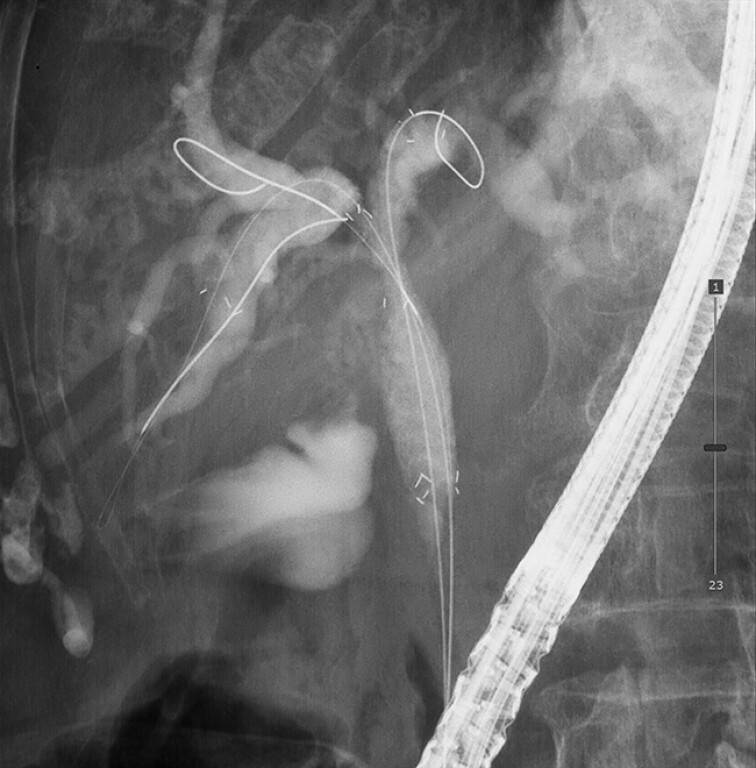
Side-by-side deployment of multi-hole-covered self-expandable metallic stents at B3 + 4 and B6 across the malignant hilar biliary obstruction.

**Fig. 4:**
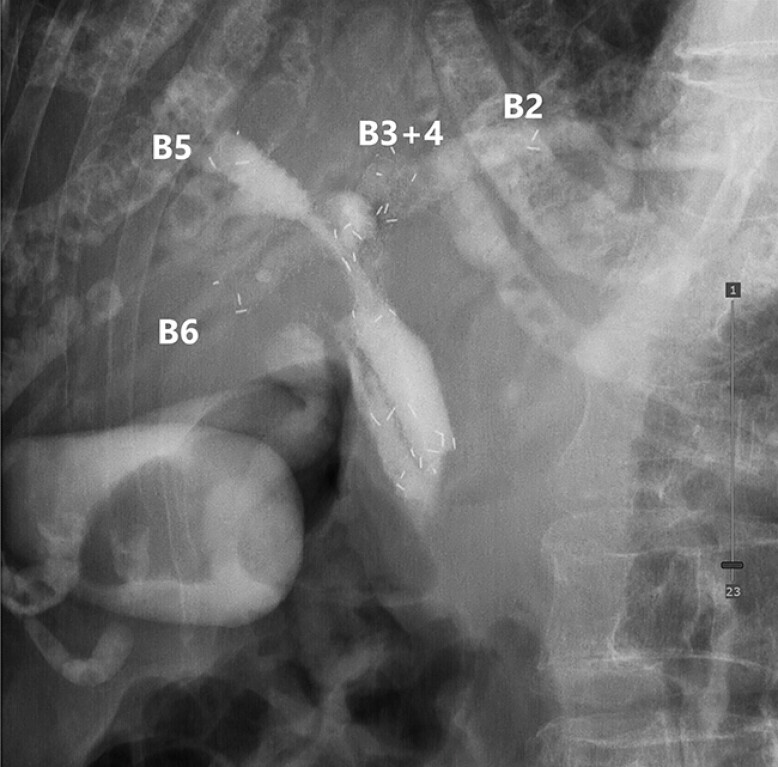
A final fluoroscopic view demonstrating successful four-branched drainage: stent-in-stent placement from the B3+4 stent to B2 and from the B6 stent to B5, resulting in four fully expanded multi-hole-covered self-expandable metallic stents.

A 91-year-old woman with bismuth type IV hilar cholangiocarcinoma initially received two inside plastic stents for the drainage of the right and left hepatic ducts. She was readmitted with acute cholangitis. Bile cultures from both drained and previously undrained intrahepatic ducts (a right hepatic duct during inside stent placement and a left hepatic duct during endoscopic nasobiliary drainage [ENBD]; Fig. 2 ) yielded methicillin-resistant Staphylococcus aureus, necessitating four-branched drainage. Intravenous antibiotics were administered before stent exchange. During ERCP, all previously placed stents and ENBD tubes were removed. Two MHSEMSs (8 mm × 6 cm and 8 cm) were then deployed at B3+4 and B6 in a SBS configuration ( Fig. 3 ). From each SBS stent, a guidewire was advanced through a side hole into the contralateral intrahepatic ducts (B2 and B5, respectively) using an uneven double-lumen cannula (PIOLAX, Kanagawa, Japan) and a radifocus guidewire (Terumo, Tokyo, Japan), which facilitated side-hole passage and enabled SIS deployment of an additional MHSEMS (8 mm × 6 cm; Video 1 , Fig. 4 ). Both technical and clinical success were achieved with no adverse events. The patient maintained an uneventful course without stent-related adverse events for 3 months.

Hybrid side-by-side and stent-in-stent placement techniques using multi-hole-covered self-expandable metallic stents for four-branched drainage in a bismuth type IV malignant hilar biliary obstruction.Video 1

These findings suggest that the MHSEMS may expand therapeutic options for advanced MHBO.

Endoscopy_UCTN_Code_TTT_1AR_2AZ
